# Transcriptional Elongation and mRNA Export Are Coregulated Processes

**DOI:** 10.4061/2011/652461

**Published:** 2011-10-05

**Authors:** Maria Micaela Molina-Navarro, Celia Pilar Martinez-Jimenez, Susana Rodriguez-Navarro

**Affiliations:** Centro de Investigación Príncipe Felipe (CIPF), Avenida Saler, 16. 46012, Valencia, Spain

## Abstract

Chromatin structure complexity requires the interaction and coordinated work of a multiplicity of factors at different transcriptional regulation stages. Transcription control comprises a set of processes that ensures proper balance in the gene expression under different conditions, such as signals, metabolic states, or development. We could frame those steps from epigenetic marks to mRNA stability to support the holistic view of a fine-tune balance of final mRNA levels through mRNA transcription, export, stability, translation, and degradation. Transport of mRNA from the nucleus to the cytoplasm is a key process in regulated gene expression. Transcriptional elongation and mRNA export are coregulated steps that determine the mature mRNA levels in the cytoplasm. In this paper, recent insights into the coordination of these processes in eukaryotes will be summarised.

## 1. Introduction

Gene expression is a coordinated multistep process that begins with transcription and RNA processing in the nucleus, followed by mRNA export to the cytoplasm for translation. In eukaryotes, it depends on the orchestrated action of several multiprotein complexes which regulate the gene expression by RNA polymerase II (RNAP-II) at multiple levels. 

Given the association of DNA with nucleosomes, they become a physical barrier for the transcription elongation by RNAP-II, where histone chaperones play an essential role in facilitating transcription *in vitro* and *in vivo,* and by establishing a bridge between RNAP-II and elongation factors (reviewed in [1]).

Interestingly, recent genome-wide profiling has provided a partial picture of the chromatin landscape, including a wide variety of epigenetic information about posttranslational modifications (PTMs), histone variants, DNA methylation patterns, and nucleosome occupancy (reviewed in [2]). In this scenario, histone turnover determines continuous access to sequence-specific DNA-binding proteins; therefore, nucleosome positioning is actively involved in gene-expression regulation. 

Mechanisms to export mRNA are integrated into the biogenesis of messenger ribonucleoparticles (mRNPs). In this context, the nuclear pore complex (NPC) provides a molecular environment that regulates gene tethering and mRNA export. Moreover, a functional connection between transcription and RNA export has been reinforced by human genetic disorders caused by mutation in mRNA export factors and adaptors [3, 4]. mRNA biogenesis requires the transport of competent mRNPs to the cytoplasm, coupling nuclear export, and formation of mature RNA. This process is complex and requires coordination between gene transcription and RNA capping, splicing, 3′-end formation, termination, and polyadenylation (reviewed in [5]).

Emerging studies have provided strong evidence for a role of the NPC in gene expression control. For instance, gene positioning is a dynamic process which determines gene regulation [6]. Actually, from the gene gating hypothesis proposed by Blobel in 1985 arises the resulting link between mRNA transcription and the NPC. Therefore, mRNA transcription and export processes are orchestrated in time and space. 

As we have seen, the complexity of chromatin structure requires the interaction and coordinated work of a multiplicity of factors at different transcriptional regulation levels. Despite the fact that proteins involved in gene expression are highly conserved from yeast to higher eukaryotes, several processes such as mRNA biogenesis, which are coupled to nuclear export, are more divergent. This paper will pay attention to the factors implicated in gene transcriptional elongation coupled to mRNA export processes, more specifically to complexes which coordinate mRNP biogenesis and whose misregulation causes important diseases.

## 2. TREX Couples Transcription Elongation and mRNA Export

Due to the compartmentalisation of the eukaryotic cell, the transcribed mRNAs need to be transported from the nucleus towards the cytoplasm to be translated into proteins. After mRNAs have been packaged into mRNPs, they can be exported. One of these proteins involved in the formation of mature mRNPs is Npl3 (9G8 and STp20 in mammals), which is an mRNA-binding protein that shuttles between the nucleus and cytoplasm for rounds of mRNPs export. Npl3 is directly involved in the mRNA export process, since mutations in this gene cause nuclear accumulation of mRNA [18, 19]. Interestingly, Npl3 was one of the first pieces of evidence in yeast to show that an export factor was required cotranscriptionally for proper mRNA export [20]. Using chromatin immunoprecipitation experiments, the Silver lab demonstrated that Npl3 binds to chromatin in a transcription-dependent manner [21]. Later, work done in different laboratories has extended this cotranscriptional regulation concept of mRNA biogenesis (reviewed in [22]). A paradigm of a complex involved in transcription elongation coupled to mRNA export is the identification of the TREX complex, which contains both factors involved in transcription elongation (THO complex) and mRNA export (Yra1 and Sub2) [5, 23, 24]. These findings allowed to put forward a first model in which TREX functions in cotranscriptional mRNP formation by promoting efficient transcription elongation through RNAP-II and by guiding the newly formed mRNP to the downstream mRNA export machinery [25]. Supporting this model, it was also found that the general mRNA export receptor can be cotranscriptionally recruited to genes and, hence, contributes to gene positioning at the NPC in an RNA-independent manner [26]. The evolutionary conservation of the TREX complex from yeast to human reveals the biological importance of the association of mRNA export and transcription components in the same complex.

The TREX complex in yeast comprises Yra1, Sub2, Gbp2, Hrb1, and the THO complex (suppressor of the transcriptional defect of Hpr1 by overexpression). The THO complex integrates the core proteins Tho2, Hpr1, Mft1, and Thp2 [23]. Among them, Yra1 (Aly/REF) is required for the export of many mRNAs [27], and it plays a key role as an mRNA adaptor factor involved in splicing-coupled mRNA export in metazoans [28, 29]. The *YRA1* gene encodes an mRNA-binding protein which acts as an adaptor for the ATP-dependent RNA-helicase Sub2 (UAP56) and is involved in splicing and mRNA export [30, 31]. Besides Yra1 and Sub2, THO interacts with other nuclear mRNA export factors, such as the SR-proteins Gbp2 and Hrb1 and Tex1 (hTex1) [24, 32, 33].

The THO complex and the aforementioned RNA export factors provide evidence for a functional link between transcription and mRNA metabolism [34]. Yra1 and Sub2 are recruited by a direct transcription-coupled mechanism as part of the TREX complex to be then transferred to nascent mRNA. In contrast, Yra1 and Sub2 accumulate poorly in a subset of intron-containing genes even though Sub2 is related to splicing, probably because the cotranscriptional spliceosome assembly inhibits their recruitment in yeast [25]. During transcription elongation, Sub2 is recruited through a direct interaction with the THO component Hpr1 [26]. Recent experiments have shown that Yra1 recruitment is Sub2 independent, but it is dependent on a direct interaction with the 3′end-processing factor Pcf11 (hPcf11), which is associated with RNAP-II early on in transcription [35]. Furthermore, the mRNA-binding protein Yra1 recruits the essential export factor Mex67 (mammalian Tap/NXF1) [36] and its partner Mtr2 (p15/NXT1) to nascent mRNA [37, 38]. The Mex67/Mtr2 heterodimer, together with Yra1, escorts the mRNP to the NPC [39]. It has been shown that Mex67 binds to the Nup84 complex, which is crucial for nuclear mRNA export [40]. Nup84 has been recently described to have a transcriptional elongation role as part of a functional NPC linked to its function in mRNA export [41]. These findings extend the role of those factors involved in mRNA transport during transcription elongation, for instance, nucleoporins. Along with Yra1 and Sub2, another Mex67 adaptor is the poly(A) binding protein Nab2, which can be transferred from the transcription machinery onto mRNA, thus ensuring that only mature mRNPs access the NPC. Nab2 is required for poly(A) tail length control and mRNA export, linking 3′end processing and export-like Npl3 [42, 43]. 

Recently, novel links between transcription elongation and mRNA export have been described. For instance, the Prp19 splicing complex (homologous to human XAB2) also has a novel function in transcription elongation, since it ensures the stabilisation of the recruitment of the TREX complex at the transcribed genes [44]. This recruitment is mediated by a component of Prp19, named Syf1, which genetically interacts with THO and is involved in both splicing and transcription-coupled DNA repair. The C-terminus of Syf1 is necessary for the interaction between the Prp19 complex and RNAP-II at the transcribed genes. Furthermore, Jimeno et al. identified two suppressors of an *hpr1Δ* mutant, Thp3 and Csn12, which form a complex that is recruited to transcribed genes, establishing a further link between transcription elongation and mRNA processing [45].

It is interesting to note how histone chaperones seem to play a key role in transcription elongation by working together with the TREX complex. Nap1 is a histone chaperone recruited to specific transcribing ORFs by the TREX component Yra1, and it is important for chromatin remodelling during transcription elongation. In addition, Nap1 shows a genetic interaction with Mex67. These data suggest a new connection between transcription elongation and mRNPs formation [46]. In human cells, a histone chaperone, Spt6, is also involved in mRNA export through its binding to the transcription elongating protein Iws1 which, in turn, triggers recruitment of REF/Yra1 to Spt6-responsive genes [47, 48]. Furthermore, UIF has been described as a novel mRNA adaptor that interacts with NXF1/Mex67 and is required for delivering mRNA to the NPC. The histone chaperone FACT, involved in transcription elongation, specifically binds UIF and is required for loading UIF onto mRNA, which ensures efficient mRNA export [49]. Accordingly, this reinforces the tight link between transcription elongation and mRNA export.

## 3. Role of TREX-2 and NPC-Regulating Gene Expression

Interaction of genes with nuclear pores also contributes to the coupling of mRNP biogenesis and export. In accordance with the “gene gating” hypothesis [50], the link between mRNA transcription and NPC export sites helps preferential processing and export of transcripts [51].

After nuclear quality control mechanisms (reviewed in [52]), mRNPs are tethered and exported to the cytoplasm. Transport of mRNA occurs through NPCs. Therefore, the NPC provides a molecular channel for the trafficking of export factors associated with their cargoes from the nucleus to the cytoplasm, and *vice versa* [53]. Nucleoporins bind to regulated genes and induce their transcription. This suggests a role of NPC components in regulating the gene expression programs in multicellular organisms [6, 54]. An example that depicts the importance of the NPC components in mRNA export is the interaction between Mlp1 and the nucleoporin Nup60, which are required for mRNA export to the cytoplasm [55]. 

An early discovery, which suggested a physical connection between the transcription machinery and the NPC, was the identification of Sus1 in yeast as a factor that is part of two complexes involved in transcription and mRNA export [56]. Sus1 was identified as an mRNA export factor in a synthetic lethal screening by using a *yra1* mutant allele [56]. It is a nuclear protein localised around the nuclear periphery through its binding to the nuclear pore-associated Sac3-Thp1-Cdc31 complex (TREX-2) involved in mRNA export [56, 57]. Sus1 is also a component of the SAGA histone acetylase complex implicated in transcription initiation. Associated with the deubiquitination module (DUBm) of the transcriptional coactivator SAGA, Sus1 is involved in chromatin modification and transcriptional activation [58].

Initially, the role of TREX-2 docking mRNPs to specific nucleoporins at the nuclear entrance of the NPC was reported. Sac3 interacts with Mex67p-Mtr2p for proper mRNA export. Furthermore, Sac3p-Thp1p TREX-2 components connect with the NPC environment through Nup1p [59]. *THP1* was originally identified as a eukaryotic-conserved gene whose null mutations conferred genetic instability, transcription defects and hyperrecombination phenotypes like those on THO mutants [60]. Recently, a new integrant of the TREX-2 complex has been found. Sem1 (Dss1 in humans) interacts physically and functionally with Thp1 and Sac3, indicating that Sem1 could be a bona-fide candidate for the TREX-2 complex [61, 62].

Most of the mutants in TREX-2 components compromise transcription elongation to different extents. Transcription elongation has been traditionally studied by genetic and biochemical approaches both *in vivo* and *in vitro*. *In vitro*, purified RNAP-II engaged directly on an oligonucleotide with a dC-tail [63], or analyses of elongation in naked DNA using whole cell extracts and plasmids with two G-less cassettes [64], have been widely used. *In vivo*, transcriptional run-on assays using a pulse of radioactive UTP and further array hybridisation [65] and RNA Pol-ChIP [66, 67] have also been extensively employed. Other assays, for instance, the GLAM ratio method, have also been successfully applied to identify factors whose mutation affects transcription elongation [68–70]. However, controversial results have been obtained depending on the assays performed, which reveal that multiple parameters might affect the specificity and yield of the factors analysed by these techniques. For instance, the role of Sus1 during transcription elongation illustrates this discordance. Several authors, including ourselves, have proposed a role for Sus1 during transcription elongation. Gonzalez-Aguilera and coworkers showed that transcription elongation is impaired in *sus1Δ* mutants *in vivo*, but only slightly *in vitro*, and they concluded that *sus1Δ* leads to similar gene expression defects as those of mutants *thp1Δ* and *sac3Δ* [71]. We demonstrated elsewhere by ChIP assays that Sus1 is recruited to coding regions during transcription elongation in association with SAGA and TREX-2 [68] and that Sus1 physically associates with export factors and with the RNAP-II. We observed that its absence elicits a decrease in total RNAP-II recruitment in pGAL-YLR454w constructs. This fits in nicely with the results obtained by the GLAM assay, which measures the efficiency of gene-length-dependent mRNA accumulation [69]. All these approaches suggest a functional role of Sus1 during transcription elongation. However, these results differ from a recent study which concluded that Sus1 does not significantly affect transcription elongation [41]. These discrepancies could be explained by antibody specificities [72, 73]. For instance, slight differences in the ChIP protocols could bring about different results due to the crosslinking effects between elongating RNAP-II and nascent mRNP [74]. It has been shown that in some studies the 8WG16 antibody specifically recognises the hypophosphorylated and, therefore, the initiating form of RNAP-II [75, 76]. Other studies, especially those in yeast and *Drosophila*, have clearly detected the elongating form of RNAP-II in chromatin immunoprecipitations [77]. However, Gilchrist et al. have demonstrated that the commonly used 8WG16 antibody has a higher affinity for initiating polymerase than for elongation-competent polymerase (hyperphosphorylated). Thus, the ChIP material derived from immunoprecipitation with 8WG16 will be inherently and substantially biased towards enrichment in the promoter-proximal RNAP-II signal, rendering this material not the most suitable for analyses of transcription elongation or RNAP-II stalling [73]. Gilchrist et al. used an antibody against the Rpb3 subunit in genome-wide analyses using ChIP-chip and ChIP-Seq to study widespread regulation of transcription elongation [73]. An antibody against Rpb3 recognises RNAP-II regardless of the phosphorylation state of the CTD of the Rpb1 subunit. Since transcription is coupled to mRNA export and the NPC plays a prominent role during this coupling, it is possible that many physiological factors account for these experimental divergences. 

Once mRNP has been properly formed and assembled, it is thought that the export is facilitated by a close location to the NPC. TREX-2 mediates the location of the active genes to the NPC through a binding to both the NPC nuclear face and the SAGA complex. The crystal structure of Sus1 and Cdc31 [16], bound to a central region of Sac3, forms a conserved interaction platform that promotes NPC association and mRNA export and provides a scaffold which integrates the interaction between protein complexes and facilitates the coupling of transcription and mRNA export [17]. Deciphering the exact TREX-2 complex structure will shed light on the mechanistic coordination of the process by the complex in response to physiological or environmental changes. 

## 4. SAGA-TREX-2 Mediates Initiation, Elongation, and Export

The SAGA complex's function in transcription activation has been largely characterised, mainly its role as a histone-modifying complex (reviewed in [78–80]). SAGA contains two enzymatic activities involved in posttranslational histone modifications: histone acetylation and deubiquitinylation [81, 82]. The SAGA chromatin-modifying complex regulates accessibility to promoter DNA in part through modification of histone amino terminal tails. Histone acetylation is one of the best-studied posttranslational modifications. SAGA contains a Gcn5-related acetyltransferase (GNAT) as a catalytic subunit. The well-established members of the complex were identified by biochemical and genetic studies first in yeast and later in *Drosophila* and humans. For simplicity reasons, the mammalian (human) GCN5-containing STAGA, TFTC, and PCAF complexes have been renamed as human SAGA by Pijnappel and Timmers in 2008 [79, 83, 84]. 

In fact, SAGA-promoted histone acetylation appears to enhance the processivity of RNAP-II during transcription elongation, indicating its direct contribution to this process [82, 85]. Furthermore, several pieces of evidence suggest a link between SAGA and mRNA export that is not only restricted to the presence of Sus1 in SAGA and TREX-2. Firstly, Sgf11 deletion enhances the mRNA export defects observed in *sus1Δ* cells [58]. Secondly, Ubp8 and Sgf73 deletions show defects in mRNA export. Hence, the interaction of Sac3 and Thp1 TREX-2 components with SAGA is crucial for mRNA export. Sgf73 appears to be a molecular scaffold which integrates regulation of H2B ubiquitination in *GAL1 *mRNA export by tethering the gene to the NPC [86]. Thirdly, Sgf73 is necessary for the association of Sus1 not only with SAGA, but also with TREX-2, which suggests that both complexes are coordinated in their role of coupling transcription to mRNA export [68]. Finally Mlp1, a protein involved in gene anchoring at the nuclear periphery, has been described to interact with SAGA and to tether the actively transcribed* GAL1* gene to the NPC [87] by expanding the links between SAGA and mRNA export.

**Figure 1:**
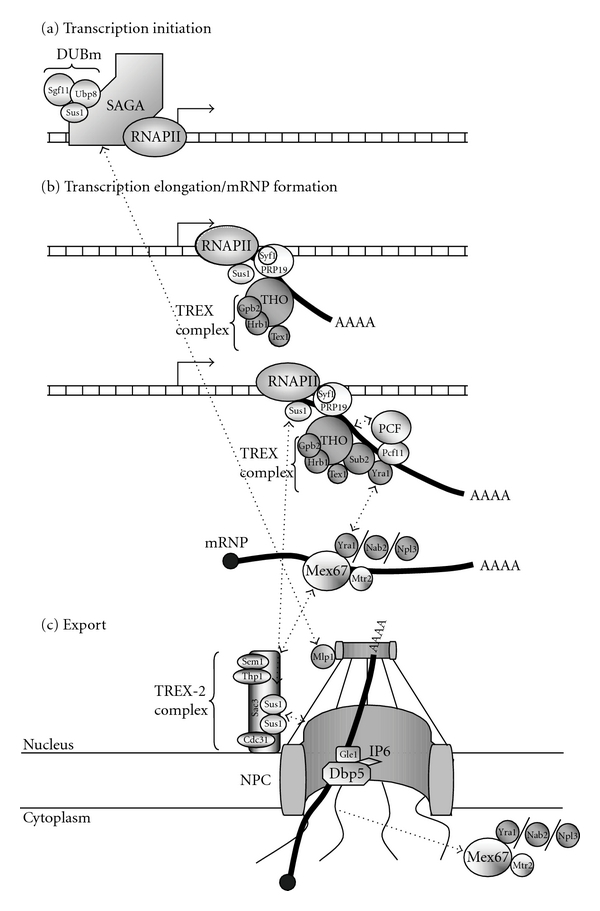
Coordination of different steps in transcription initiation, mRNP biogenesis, and export facilitates gene expression. (a) Active genes are recruited to the nuclear periphery through the factors involved in both transcription and mRNPs export. In the transcription activation process, there is an interaction via SAGA and Sus1 between the promoter and the NPC tethering the genes to the nuclear periphery. Mlp1 (myosin-like protein 1) is also involved in the recruitment of the *GAL1* gene to the NPC upon transcription activation. (b) The TREX complex is recruited to nascent mRNA in the early steps of transcription elongation although there are differences between yeast and metazoan when considering genome complexity. Whereas TREX is recruited cotranscriptionally in yeast, it is recruited by spliceosome components in metazoan likely due to the highest percentage of intron-containing genes [14, 15]. Adaptor proteins (Yra1, Nab2, and Npl3) recognise RNA when the transcript is competent for export to the cytoplasm, and they recruit it to export factors such as Mex67-Mtr2. This recruitment to export factors is crucial for generating mature mRNP. (c) Once the mRNP is properly formed and assembled, it is assumed that the export is facilitated by a close location to the NPC. TREX-2 (also known as THSC) mediates the location of active genes to the NPC through a binding to both the NPC nuclear face and the SAGA complex. The crystal structure of Sus1 and Cdc31 [16], bound to a central region of Sac3, forms a conserved interaction platform that promotes NPC association and mRNA export to provide a scaffold that integrates the interaction between protein complexes and facilitates the coupling of transcription and mRNA export [17]. Thp1 and Sac3 mediate the docking of mRNP at the NPC through its interaction with Mex67-Mtr2 and nucleoporins. mRNPs share the 5′-to-3′ polarity of movement through the NPC. Although the exact manner of how the transport of mRNPs takes place has not been precisely described, the most widely accepted hypothesis is that mRNPs are pulled through the NPC via ATP hydrolysis by the shuttling ATPase Dbp5 (hDbp5). Dbp5/Rat8 binds to the cytoplasmic filaments of the NPC by interacting with two nucleoporins (Nup159 and Nup 42) and Gle1. Gle1, together with its cofactor IP6, stimulates the ATPase activity of Dbp5. Once inside the cytoplasm, mRNA is released, and mRNP proteins are removed by entering a new export cycle.

Several nuclear steps in mRNPs formation occur in close temporal and physical proximity to the NPC. In this scenario, SAGA and TREX-2 could coordinate the expression of a subset of genes in the vicinity of the NPC. The precise sequence of events is poorly understood, but a consensus model for the mechanism of this process is framed by the Blobel gene-gating hypothesis [50]. According to this model (Figure 1), the SAGA chromatin remodelling complex is recruited to the promoter of a subset of inducible genes and is needed for their transcription. As mentioned above, components of the TREX-2 complex interact with SAGA. The Sus1 protein, a member of the SAGA DUBm, facilitates the interaction between SAGA and TREX-2 components, which conducts a relocation of transcriptionally active genes to the nuclear periphery. Hence, the gene-gating process triggers the loading of mRNA export factors on the nascent transcript. In a subsequent step, the THO complex might interact with the new generated mRNPs throughout Sub2 and Yra1 by transferring mRNA to Mex67 and Mtr2, resulting in the formation of an export-competent mRNP. It is currently accepted that other adaptors, such as Nab2 and Npl3, are players in the process and are determinants in the association of mature mRNPs with the NPC. Moreover, the presence of Sus1 in coding regions and its physical interaction with RNAP-II imply that Mex67 and Yra1 are suggestive of a further role of Sus1 in downstream events during mRNA biogenesis [68]. Subsequently, the THO complex is released from the transcription site before transcription termination occurs [88], and Sub2 association with Sac3p establishes a physical link between THO and TREX-2 [24], whence TREX-2 is involved in mRNA export with a role on the nuclear side of the NPC. 

Besides Gcn5-mediated histone acetylation, several studies have demonstrated that K123 at the C-terminus of H2B is ubiquitylated (ubH2B). ubH2B is essential for the trans-tail methylation of histone H3 and is also required for optimal gene activation [81]. Thus, histone modification marks could determine the regulatory mechanisms in transcription elongation coupled to export. Strikingly, deletions in both the *UBP8* and *GCN5 *genes responsible for SAGA enzymatic activities cause synergistic transcription elongation defects, suggesting that the roles of histone acetyl transferase (HAT) and DUB modules in elongation are functionally distinct [89]. Among the epigenetic marks, ubiquitination of histone H2B, therefore, regulates chromatin dynamics by enhancing nucleosome stability. It is interesting to speculate that histone H2B deubiquitination might be of special relevance in the promoters recruited to the NPC via the SAGA-TREX-2 connection. Further work is required to determine this intriguing possibility.

All-encompassing, these models have been established from the different experimental and structural data obtained in *S. cerevisiae*. Nevertheless, several pieces of evidence open up the possibility that gene tethering to the nuclear periphery might also occur in some cases in other eukaryotes (reviewed in [90]). Interestingly, upon the inactivation of two NPC components, the transcription of the X-linked genes reduces, suggesting that the connection between the NPC and gene activation might be a conserved mechanism in eukaryotes [91]. 

## 5. mRNA Biogenesis and mRNP Export Defects Leads to Diverse Human Disorders

Nuclear factor export proteins, along with NPC components, play a critical role in the selective transport of proteins, RNA and ribonucleoproteins across the nuclear envelope. Defects in mRNA export, as well as Nups mutations, have been linked to several human diseases Table 1. Interestingly, the observed phenotypes are often manifested in specific cell types and in particular molecular pathways [3, 4, 6–8, 92]. For instance, a dynamic and stable transcription of Nups takes place in dividing *versus* terminally differentiated cells. D'Angelo et al. showed in 2009 that lack of replacement of NPC scaffold components in the somatic cells from *C. elegans* and rat brain neurons comes with an age-dependent deterioration of the NPC, leading to an aberrant nuclear accumulation of cytoplasmic tubulin [93]. In humans, there is a strong tissue-specific requirement for Nups, which are associated with specific pathologies (reviewed [6]). One example that depicts Nups requirements is atrial fibrillation (AF) caused by a mutation in the human *NUP155* which, in turn, can lead to sudden cardiac death [7]. Experiments with heterozygous Nup155^-/+^ mice have shown an AF phenotype, suggesting that reduction in the level of *NUP155,* or its mistargeting, results in a tissue-specific disorder [12]. Other examples are the translocations in *NUP98* and *NUP214* genes, which have been characterised as mutations leading to several types of leukaemia [8–10].

Furthermore, the human TREX complex has been identified as a culprit of aggressive human breast cancer [13]. hTREX84, a subunit of the hTREX complex, is highly expressed in this kind of cancer, and its expression is strongly associated with an aggressive phenotype of human breast tumours [13]. Hence, Guo et al. identified not only hTREX84 as a prognosticator of breast cancer, but also the delineated human TREX complex as a target for therapeutic drugs against breast cancer [13]. 

Several lines of experiments in yeast have proposed Sus1 (ENY2 orthologue) to be a mediator between the NPC, Nups, and active genes [56] and also a link between transcription and mRNA export [68]. Sus1 plays a critical role in the modularity of the DUBm, which is conserved in *Drosophila* and humans. Thus, we could also expect that defects in DUBm composition would affect mRNA export. Therefore, mRNA processing defects could trigger diverse genetic disorders Table 1. Indeed, the interaction between DUBm and the SAGA complex has been shown to be mediated by TAF5L and ATXN7 [94]. Interestingly a decade ago, ATXN7 was correlated with neurodegenerative disease spinocerebellar ataxia type 7 (SCA7) [11]. ATXN7 is an integral component of SAGA [95], and several studies in yeast have demonstrated that the yeast orthologue ATXN7 (Sgf73) anchors DUBm to the SAGA complex [86]. Mutations in Sgf73 result in a release of DUBm from SAGA [56, 86]. These data open up several possibilities, for instance, (i) SAGA-independent DUBm could regulate a subset of target genes, (ii) DUBm could determine the recruitment of SAGA in particular gene promoters, (iii) or even a combination of both.

Further studies are needed to shed light on the molecular mechanism underlying the specificity and the biological role of mRNA export factors, TREX components, and NPC coupling transcriptional initiation, elongation, and mRNA export of *in vivo* targets. Likewise, understanding the time-specific interaction of these components during development will unravel the tight-coupled mechanism that regulates the cytoplasmic fate or their target mRNAs. 

## 6. Concluding Remarks

The latest advances in proteomics have improved knowledge about the composition of protein complexes, and the newly established protein-protein networks show additional levels of plasticity to coordinate mRNA transcription, elongation, and export. However, further studies are needed to comprehend the particular contribution of each component orchestrating the gene expression in eukaryotes. 

Deciphering the molecular mechanisms that coordinate the transcriptional output and mRNA level in response to cellular signals will be a future determinant to discover new therapeutic targets and new cellular pathways involved in different processes, for example, development and differentiation in response to stress conditions or even in complex diseases such cancer and human genetic disorders. 

## Figures and Tables

**Table 1:** Human disorders associated with mRNA biogenesis and mRNA export defects.

Gene	Pathologies/disorders	Comments	References
GLE1	Lethal congenital contracture syndrome 1 (LCCS1)	Encodes a protein required for the export of mRNAs from the nucleus to the cytoplasm and is critical in motoneuron development and maturation.	[3]

X-linked mental retardation (XLMR)	Fragile X syndrome (FXS)	Inactivation of the X-linked FMR1 gene leads to the loss of its encoded protein FMRP and RNA export factor NXF2, causing defects in neuronal development and function as well as in male germ cells.	[4]

NUP155	Atrial fibrillation (AF)	Mutations in the gene are an inherited form of clinical arrhythmia that can lead to sudden cardiac death.	[7]

NUP98 and NUP214	Acute myelogenous leukemia (AML).	Translocations in the gene have been characterised as mutations leading to several types of leukaemia.	[8–10]

ATXN7	Neurodegenerative disease spinocerebellar ataxia type 7 (SCA7)	The expansion of an unstable CAG repeat in the first exon of the SCA7 gene causes this neurodegenerative disease	[11]

USP22	Associated with poor prognosis of diverse cancer types	Catalyses the deubiquitylation of histone H2B and is required for appropriate cell-cycle progression. Component of the 11-gene polycomb/cancer stem-cell signature	[12]

TREX84	Breast cancer	Its expression is strongly associated with an aggressive phenotype of human breast tumour	[13]
